# Longitudinal Changes in Refractive Error Among Preschool Children Aged 1–6 Years: The Changsha Children Eye Study

**DOI:** 10.3389/fmed.2022.831177

**Published:** 2022-03-23

**Authors:** Yuxia You, Ming Xu, Yali Song, Huanfen Zhou, Shihui Wei

**Affiliations:** ^1^Department of Ophthalmology, The Chinese People's Liberation Army Medical School, The Chinese People's Liberation Army General Hospital, Beijing, China; ^2^Beijing Aier Intech Eye Hospital, Beijing, China; ^3^Hunan Super Vision Technology Co., Ltd., Changsha, China

**Keywords:** refractive error, preschool myopia, myopia, risk factors, retrospective cohort studies

## Abstract

**Purpose:**

To investigate the longitudinal changes in refractive error of preschool children and explore the factors related to these changes and the timing of intervention.

**Methods:**

The refractive data of preschool children aged 1–6 years were collected from 16 community Health Service Centers in Changsha during April 2016 to July 2019 for the retrospective cohort study. The refractive data of each participant was measured with a hand-held vision screener without cycloplegia. A follow-up for all the included participants was performed. The spherical equivalent change was calculated, subsequently, an analysis of risk factors related to the change was performed.

**Results:**

Four thousand nine hundred twenty-one cases were included in the study with the follow-up for 1–2 years. The refractive status was found smoothly changed in 67.8% of children. The overall initial SE was 0.62 ± 1.13 D, and the average SE change was −0.20 ± 1.23 D per year. However, profound myopic shift was observed in 32.2% of children. The change of SE in 3-year-old group is most overt. The proportions of 1–6 years old who showed moderate and severe myopic shift (SE change ≥–1.00 D) were 21.6, 18.9, 28.2, 25.5, 13.4, and 10%, respectively. At the first visit, the younger children with greater hyperopic state exhibited more noticeable myopic shift, no significant difference was found in gender.

**Conclusion:**

The shift from hyperopia to myopia in preschool children is smooth, with −0.20D change on average per year. We suggest that an optometry screening should start at 3-year-old to track children's refractive status. We recommend that preschool children whose SE changes more than −1.00 D per year go to the ophthalmology department for further examination. Our study also found that at the first visit, the younger the child is and the more positive initial SE is, the degree of shift of myopia is greater.

## Introduction

Myopia is an underrated but profound public health problem, which brings enormous potential economic impact (1). In 2015, uncorrected refractive errors were estimated to be the leading cause of moderate or severe visual impairment, affecting over 116 million people (2), and the global economic burden associated with uncorrected myopia was estimated to be 244 billion U.S. dollars (3). Furthermore, it is estimated that nearly half of the worldwide population will be myopic, including 10% high myopia by 2050 (4).

Among some of young adults in Asia, the prevalence of high myopia is 38% (5). At present, the prevalence of myopia in China remains high, with 60% in 12-year-old primary school graduates, around 80% in 16-year-old high school students, and more than 90% in college students (6–8). Myopia has brought a substantial economic burden to China. Reference to the direct cost of myopia in Singapore children is US$148 per child per year (9).

Myopia mainly occurs in school-age children over 6 years old (10), and the prevalence of it under 6 years old, which is called preschool myopia, is relatively low (11–14). However, in preschool myopia, the risk of developing into high myopia and secondary related irreversible blinding complications are higher (15, 16). Since myopia is irreversible once it occurs, we should move the prevention of myopia ahead of time and pay attention to the refractive status and its' change of preschool children to better control the progress myopia.

Although the government and the general public are paying more attention to myopia, the ophthalmologists and optometry are too few to provide the professional ophthalmic care to all patients with myopia. Nowadays, community health service centers undertake most of primary eye care services, for instance vision screening for preschool children aged 1–6 years. To check the refraction status of children, the general practitioners in community health service centers usually use a hand-held vision screening equipment which is portable, convenient, easy to use, quick in inspection, and the results are intuitive and easy to interpret. For optical status inspection, many studies have also confirmed that the test results are highly consistent with the previous retinoscopy and computer refractor (17).

For vision screening, cycloplegia is not routinely applied, unless a child is known to have abnormal refractive error. Except for children with abnormal vision acuity, preschool children with what kind of refractive state should go to the ophthalmology department for further dilated refraction? There is no definite conclusion yet. Until now, there are few cohort studies on the refractive status of preschool children, especially the lack of extensive sample studies on the refractive status of children before 3 years old. Therefore, in cooperation with the community health center in Changsha, we conducted a retrospective cohort study about the changes in the refractive status of preschool children aged 1–6 years and explored the factors related to these changes and the timing of intervention.

## Subjects and Methods

### Research Object

The Changsha Children Eye Study (CCES) is a population-based study of Chinese children to estimate the prevalence and risk factors for refractive errors and ocular diseases. This study was approved by the ethics committee of Beijing Aier Intech Eye Hospital and performed from April 2016 to July 2019 among children aged 1–6 years from 16 communities in Changsha, China. The data were obtained through the Mulin telemedicine platform (Hunan Super Vision Technology Co., Ltd.).

### Inclusion and Exclusion Criteria

Inclusion criteria: (1) Preschool children aged 1–6 years. According to the child's date of birth, children under 6 years old were included in this study on the examination day. Six groups were generated by age: 1-year-old group (child with age ≤ 1-year-old on the examination day), 2-year-old group (1 year < age on the examination day ≤ 2 years), 3-year-old group (2 years < age on the examination day ≤ 3 years), 4-year-old group (3 years < age on the examination day ≤ 4 years), 5-year-old group (4 years < age on the examination day ≤ 5 years), 6-year-old group (5 years < age on the examination day ≤ 6 years).

Exclusion criteria: (1) Children with systemic cardiovascular diseases, such as congenital heart disease. (2) Children with eye trauma or eye diseases, such as congenital glaucoma, congenital cataract, strabismus. (3) Children with incomplete electronic medical records.

### Examination Method

All children who came to the community for child health checkups were invited to participate in vision screening. After the consent of participating the study were obtained from children's parents or their legal guardians. The community doctors who were trained in standardized procedures would ask about the history of childhood systemic diseases and eye diseases and exclude children with systemic diseases and congenital eye diseases such as glaucoma and cataracts. The cover-uncover test was performed to exclude children with strabismus.

A handheld child vision screener Suowei (Tianjin Suowei Electronic Technology Co., Ltd.), was used to screen children's binocular refractive condition. The vision screener was calibrated daily before the testing. Children underwent routinely examinations without cycloplegia in a dark room by a general practitioner. Before the study, all the general practitioners were trained by ophthalmologists in terms of conducting standard eye examination and using the handheld child vision screener. The binocular spherical, astigmatism, astigmatism axis, pupil size, pupilary distance, and fixation direction were obtained, recorded, and uploaded on the Mulin telemedicine platform.

### Diagnostic Criteria

Spherical equivalent (SE) is calculated by the sphere plus half of astigmatism. The main result of this study is the change of SE, which is the difference between the final SE and the initial SE to represent the change in the refractive error of each child. It is defined that the change of SE(ΔSE) exceeds 0.50 D (Diopter, D) as the shift of myopia. In our study, four criteria ( ≤ −0.5D, ≥0.50 D, ≥−1.00 D, ≥−2.00 D) was used to classify the degree of shift of myopia (no change, mild shift of myopia, moderate shift of myopia, and severe shift of myopia).

### Statistical Analysis

Statistical analysis was performed using SPSS software (IBM-SPSS, V 20.0). In addition to general descriptive statistics, paired *T*-test, one-way ANOVA, and logistics regression were used to analyze data. *P* < 0.05 is considered statistically significant.

## Results

### Characteristics of Data

This study included 4,921 preschool children aged 1–6 who completed 1–2 years of follow-up in 16 community health service centers in Changsha from April 9, 2016, to July 30, 2019, of which 2,571 (52.25%) were in 1-year-old group, 392 cases (7.97%) were in 2-year-old group, 756 cases (15.36%) were in 3-year-old group, 916 cases (18.61%) were in 4-year-old group, 276 (5.61%) cases were in 5-year-old group , and 10 cases (0.20%) were in 6-year-old.

### Initial Refractive Error

**Figure 1:**
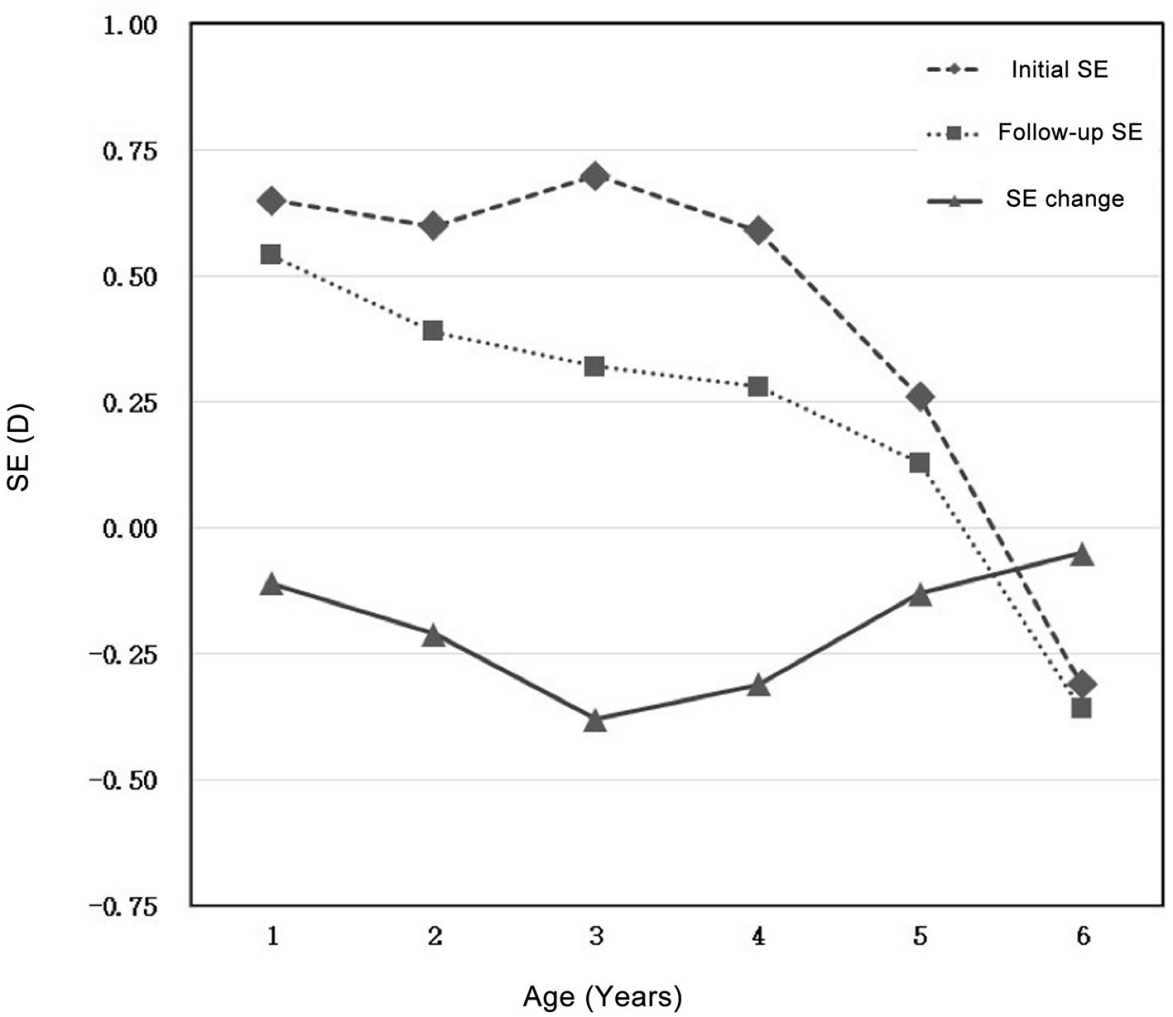
Initial SE, follow-up SE, and SE change for preschool children aged 1–6 years.

**Figure 2:**
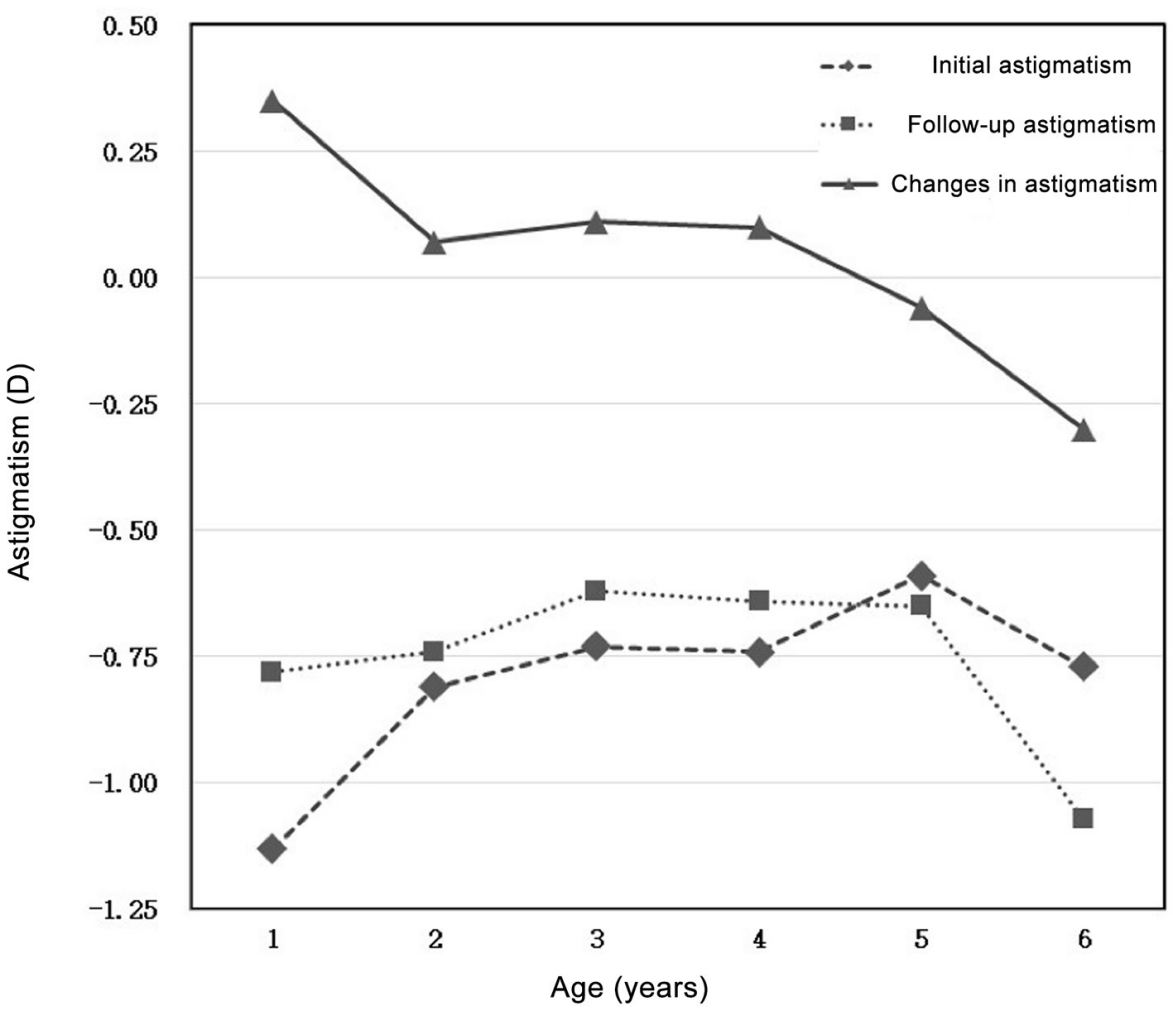
Initial astigmatism, follow-up astigmatism, and changes in astigmatism in preschool children aged 1–6 years.

The average SE of 1–6 years old preschool children is 0.62 ± 1.13 D for the right eye and 0.71 ± 1.18 D for the left eye; the average astigmatism of the right eye is −0.94 ± 0.75 D, and the left eye is −0.95 ± 0.75 D (Table 1). Among the groups, with the increasing of age, the SE decreased and slightly shifted to myopia (Figure 1); astigmatism decreased from 1 to 5 years old, among which astigmatism decreased significantly at 1–2 years old and then stayed relatively stable (Figure 2).

**Table 1:** The initial refractive error of preschool children aged from 1 to 6-year-old.

**Age**	**Number**	**Right eye**		**Left eye**	
**(years)**		**SE**	**DC**	**SE**	**DC**
1	2,571	0.65 ± 1.25	−1.13 ± 0.80	0.74 ± 1.27	−1.13 ± 0.80
2	392	0.60 ± 1.07	−0.81 ± 0.70	0.67 ± 1.14	−0.85 ± 0.70
3	756	0.70 ± 1.04	−0.73 ± 0.59	0.80 ± 1.10	−0.74 ± 0.58
4	916	0.59 ± 0.99	−0.74 ± 0.64	0.68 ± 1.06	−0.73 ± 0.63
5	276	0.26 ± 0.75	−0.59 ± 0.53	0.44 ± 0.98	−0.68 ± 0.57
6	10	−0.31 ± 0.34	−0.77 ± 0.73	−0.28 ± 0.34	−0.95 ± 0.48
Total	4,921	0.62 ± 1.13	−0.94 ± 0.75	0.71 ± 1.18	−0.95 ± 0.75

*SE, spherical equivalent; DC, cylinder degree*.

### Follow-Up Refractive Error

After 1–2 years of follow-up, the average SE of preschool children aged 1–6 years is 0.43 ± 0.93 D for the right eye and 0.47 ± 0.96 D for the left eye; the average astigmatism of the right eye is −0.72 ± 0.62 D, and the left eye is −0.71 ± 0.63 D (Table 2). Similarly, with the increasing of age, the average SE decreased and shifted toward myopia among age groups (Figure 1). Whereas, astigmatism was approximately stable among all age groups (Figure 2).

**Table 2:** Follow-up refractive error of preschool children aged from 1 to 6-year-old.

**Age**	**Number**	**Right eye**		**Left eye**	
**(years)**		**SE**	**DC**	**SE**	**DC**
1	2,571	0.54 ± 1.01	−0.78 ± 0.63	0.58 ± 1.01	−0.76 ± 0.64
2	392	0.39 ± 0.88	−0.74 ± 0.67	0.36 ± 0.91	−0.73 ± 0.68
3	756	0.32 ± 0.83	−0.62 ± 0.56	0.42 ± 0.90	−0.62 ± 0.59
4	916	0.28 ± 0.83	−0.64 ± 0.61	0.34 ± 0.90	−0.63 ± 0.61
5	276	0.13 ± 0.53	−0.65 ± 0.61	0.22 ± 0.65	−0.74 ± 0.63
6	10	−0.36 ± 0.62	−1.07 ± 0.80	−0.39 ± 0.65	−1.05 ± 0.86
Total	4,921	0.43 ± 0.93	−0.72 ± 0.62	0.47 ± 0.96	−0.71 ± 0.63

*SE, spherical equivalent; DC, cylinder degree*.

### Changes in Refractive Error

To calculate the changes of refractive errors, ΔSE and ΔDC was calculated by subtracting the initial SE and initial astigmatism by mean SE and mean astigmatism measured at the end-point of follow-up, respectively. The average ΔSE of preschool children aged 1–6 years are −0.20 ± 1.23 D (right eye) and −0.24 ± 1.26 D (left eye); the average ΔDC of preschool children aged 1–6 years are: 0.22 ± 0.73 D (right eye) and 0.23 ± 0.72 D (left eye) (Table 3). Compared with mean value of initial SE and astigmatism, both of mean SE and mean astigmatism measured at the end-point of follow-up decreased. with the most obvious decrease (−0.38 ± 1.22 for right eye, −0.37 ± 1.24 for left eye) found in 3-year-old group. Astigmatism changes more obviously in the 1-year-old group (0.35 ± 0.81 for right eye, 0.37 ± 0.80 for left eye), and changes slightly in other age groups (Figures 1, 2).

**Table 3:** Changes in refractive error of preschool children aged from 1 to 6-year-old.

**Age**	**Number**	**Right eye**		**Left eye**	
**(years)**		**Δ SE**	**Δ DC**	**Δ SE**	**Δ DC**
1	2,571	−0.11 ± 1.31	0.35 ± 0.81	−0.16 ± 1.32	0.37 ± 0.80
2	392	−0.21 ± 1.08	0.07 ± 0.70	−0.31 ± 1.10	0.11 ± 0.66
3	756	−0.38 ± 1.22	0.11 ± 0.57	−0.37 ± 1.24	0.12 ± 0.56
4	916	−0.31 ± 1.16	0.10 ± 0.57	−0.34 ± 1.22	0.10 ± 0.54
5	276	−0.13 ± 0.79	−0.06 ± 0.55	−0.22 ± 0.85	−0.05 ± 0.55
6	10	−0.05 ± 0.52	−0.30 ± 0.47	−0.11 ± 0.50	−0.10 ± 0.54
Total	4,921	−0.20 ± 1.23	0.22 ± 0.73	−0.24 ± 1.26	0.23 ± 0.72

*Δ SE, the change of spherical equivalent; Δ DC, the change of cylinder degree*.

There was no statistically significant difference in the change of binocular SE (t = 2.454, *P* = 0.117); the change of binocular astigmatism was not statistically significant (t = 3.113, *P* = 0.078).

**Figure 3:**
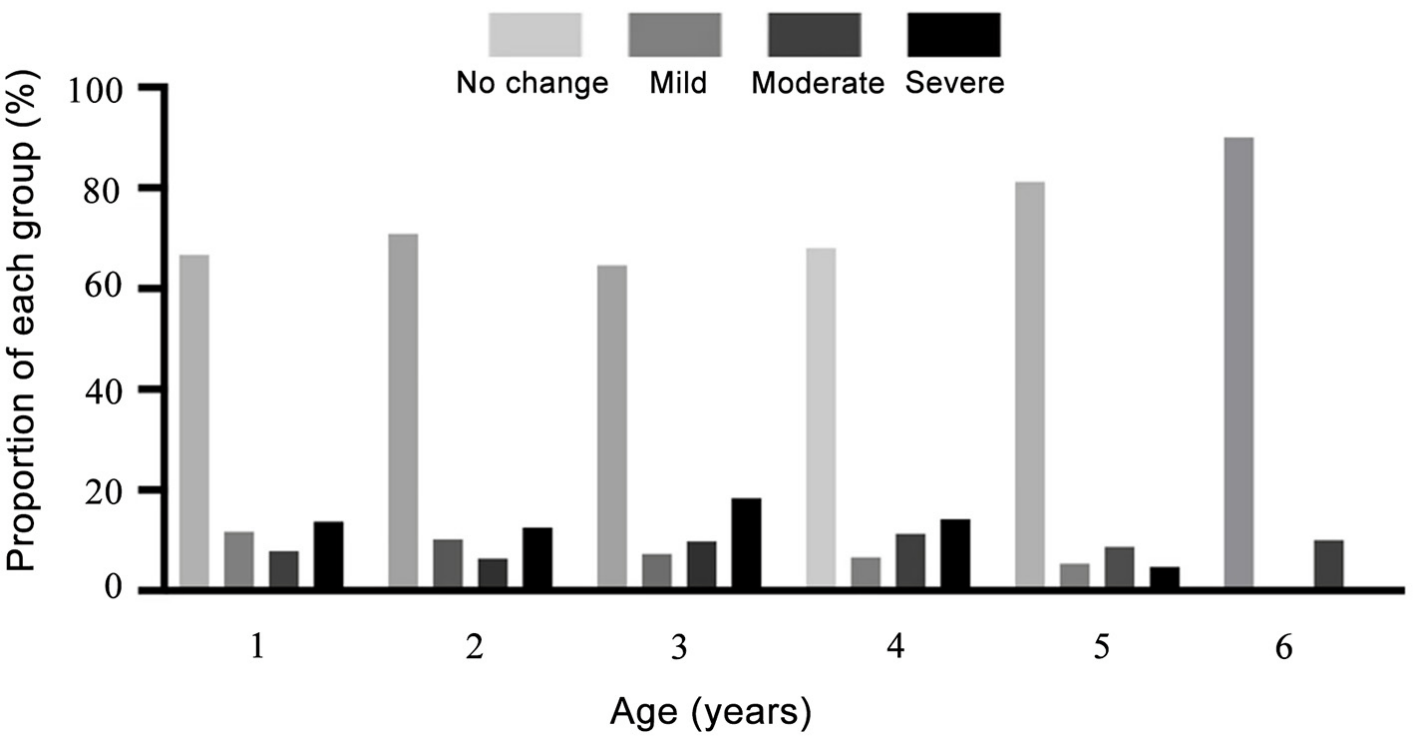
The proportions of each group in myopia progression classification for preschool children aged 1–6 years.

For the degree of shift of myopia, generally 67.8% (3335/4921) of preschool children present SE change ≤ −0.5 D, 32.2% (1586/4921) of children exhibit variable degrees of myopic drift. Similar tendency was found in all age groups (Figure 3). Notably, in 3-year-old group, 18.4% children were found with sever shift of myopia, which is distinctly higher than others (Table 4).

**Table 4:** Classification of the degree of shift of myopia in preschool children aged from 1 to 6-year-old.

**Age (years)**	**Number**	**No change**	**Mild**	**Moderate**	**Severe**
1	2,571	1,714 (66.7%)	302 (11.7%)	203 (7.9%)	352 (13.7%)
2	392	278 (70.9%)	40 (10.2%)	25 (6.4%)	49 (12.5%)
3	756	488 (64.6%)	55 (7.3%)	74 (9.8%)	139 (18.4%)
4	915	622 (68.0%)	60 (6.6%)	103 (11.3%)	130 (14.2%)
5	276	224 (81.2%)	15 (5.4%)	24 (8.7%)	13 (4.7%)
6	10	9 (90.0%)	0 (0%)	1 (10.0%)	0 (0%)

### Analysis of Factors Related to Changes in Refractive Error

Logistic regression was used to analyze the factors related to the change of SE. After the statistical test, χ^2^ = 13.951, *P* = 0.003, and the logistic regression model are significant.

The change of the SE exceeds −2.00 D, that is, the shift of myopia exceeds 2.00 D in 684 cases, which are related to age and the initial SE (*P* = 0.000; *P* = 0.000), not related to gender and initial astigmatism (*P* = 0.508; *P* = 0.429). The change of the SE exceeds −1.00 D in 1,114 cases, which are related to age and the initial SE (*P* = 0.000; *P* = 0.000) but are not related to gender and initial astigmatism (*P* = 0.139; *P* = 0.775). The change of the SE exceeded −0.50 D in 1,586 cases, which were related to gender, initial SE, and initial astigmatism (*P* = 0.021; *P* = 0.000; *P* = 0.000), Not related to age (*P* = 0.094) (Table 5).

**Table 5:** Correlation analysis for the risk factors and the change of SE.

**Factors**	**Δ** **SE**		
	**> −0.50 D**	**> −1.00 D**	**> −2.00 D**
Age	0.094	0.000	0.000
Gender	0.021	0.139	0.508
Initial SE	0.000	0.000	0.000
Initial DC	0.000	0.775	0.429

*Δ SE, the change of SE*.

## Discussion

Most infants are with hyperopia. As they grow up, the degree of hyperopia graudually declines until emmetropization completes or even develops into myopia (18–20). The refractive error of preschool children has been reported by different studies (21–24), but the sample numbers are relatively small, and most of them are cross-sectional studies. In this study, we carried out a retrospective cohort study about the longitudinal changes of 4,921 preschool children aged 1–6 years from the Changsha Community Health Service Center. All the participants were followed up for 1–2 years to analyze the changes of refractive status. Correlation of related factors which may contribute to the changes were analyzed.

The initial mean SE of all participants was 0.62 ± 1.13 D (right eye), after 1–2 years of follow-up, the value decreased by 0.20 ± 1.23 D (right eye), indicating a tendency of emmetropization. For majority of children in different age groups, the degree of change was slight (ΔSE ≤ 0.5D) with minor changes in astigmatism. The initial average astigmatism of right eye of all participants was −0.94 ± 0.75 D, after 1–2 years it dropped by 0.22 ± 0.73 D. In general, astigmatism shows a downward trend with the increasing age, and the 1-year-old group presents the most apparent decline.

Different cohort studies (21, 25, 26) reported that the degree of shift of myopia per year of school-age children was 0.39–0.68 D. In this study, preschool children's shift of myopia is 0.20 D in 1–2 years, which is lower than the research (5) (0.59 D) in Guangzhou. Most school-age children have myopia progression after myopia develops, while for preschool children, shift of myopia is the process of emmetropia. Our previous cross-sectional study found that the prevalence of myopia in preschool children decreases with age (unpublished). With age, the distribution of refractive status appears to be more concentrated toward the average. Therefore, when calculating the average change in SE of all preschool children, this offset from myopia to the emmetropia makes the average SE change smaller than the school-age myopia progression.

Many studies of school-age children (5, 27, 28) found that gender is a risk factor for myopia. The Beijing Children's Eye Disease Study (29) also showed that females and older age are high-risk factors for myopia. Shandong Children's Eye Disease Study (7), which includes mainly school-age children and some pre-school children, also found that females and older age are risk factors for myopia. Our study found that myopia drift is not related to gender. We presume that school-age girls spend more time studying and have fewer outdoor activities than boys, but the two behaviors are not significantly different at the preschool age.

However, some cohort studies (30, 31) showed that the younger the age at the first follow-up is, and the more negative the SE is, the faster myopia progresses. Our study found that at the first visit, the younger children with greater hyperopic state exhibited more noticeable myopic shift. Our findings are consistent with many reports (25, 32, 33), but the Guangzhou preschool myopia cohort study found that older preschool children and children with lower negative SE at the first visit showed higher myopia progression. It may be considered that the children they enrolled in the group were already myopic at the beginning, which is more similar to the progression of school-age myopia.

In this study, the most apparent change in SE occurred at the age of three, which may be related to the beginning of kindergarten. We recommend that regular optometry examinations should be started at this time. For a child with SE change more than 1.00 D per year, we recommend the child to go to the ophthalmology department for further examination and track the changes in axial length, and if necessary, to determine the refraction degree after cycloplegia.

The limitations of this study are (1) This is a retrospective cohort study, so other related factors such as parents' refractive status, outdoor activities and light intensity, and other lifestyle differences may not be well controlled. (2) Failure to use cycloplegia drugs may result in relative inaccurate refractive error. Although our main observation index is the change in SE, there may still be deviations. (3) Although many studies have reported that automated refraction and retinoscopy are highly correlated (34), there are still minor differences. Therefore, there may be a small deviation when we use children's vision screeners to obtain data. (4) The young age group, especially the infants in the 1-year-old group, had poor cooperation which might cause some deviations in corresponding data.

In general, our research studied the changes of refractive status of 4,921 preschool children aged 1–6 years old in 1–2 years, and found that there was a stable shift from hyperopia to myopia, with −0.20D change on average per year. Since the change of SE in 3-year-old group is most overt, we suggest that an optometry screening should start at this age to track children's refractive status. We recommend that preschool children whose SE changes more than −1.00 D per year go to the ophthalmology department for further examination. As age increases, astigmatism also shows a downward trend. Our study also found that at the first visit, the younger the child is and the more positive initial SE is, the degree of shift of myopia is greater. The change of SE is not related to gender. More prospective studies need to be carried out, such as changes in ocular biological parameters, to better explain the factors related to changes in preschool refractive status and to better prevent and control myopia in preschool children.

## Data Availability Statement

The original contributions presented in the study are included in the article/supplementary material, further inquiries can be directed to the corresponding author/s.

## Ethics Statement

The studies involving human participants were reviewed and approved by Beijing Aier Intech Eye Hospital's Ethics Committee. Written informed consent to participate in this study was provided by the participants' legal guardian/next of kin.

## Author Contributions

SW and YY designed the experiment. YY, YS, MX, and HZ performed the experiment. YY wrote the paper. All authors contributed to the article and approved the submitted version.

## Conflict of Interest

YS and MX were employed by the company Hunan Super Vision Technology Co., Ltd. The remaining authors declare that the research was conducted in the absence of any commercial or financial relationships that could be construed as a potential conflict of interest.

## Publisher's Note

All claims expressed in this article are solely those of the authors and do not necessarily represent those of their affiliated organizations, or those of the publisher, the editors and the reviewers. Any product that may be evaluated in this article, or claim that may be made by its manufacturer, is not guaranteed or endorsed by the publisher.
